# Insights into Plant Programmed Cell Death Induced by Heavy Metals—Discovering a *Terra Incognita*

**DOI:** 10.3390/cells10010065

**Published:** 2021-01-04

**Authors:** Klaudia Sychta, Aneta Słomka, Elżbieta Kuta

**Affiliations:** Department of Plant Cytology and Embryology, Institute of Botany, Faculty of Biology, Jagiellonian University in Kraków, 9 Gronostajowa Str., 30-387 Kraków, Poland; aneta.slomka@uj.edu.pl (A.S.); elzbieta.kuta@uj.edu.pl (E.K.)

**Keywords:** programmed cell death, necrosis, abiotic stress, heavy metal stress, cell culture, gene expression, plant PCD vs. animal PCD

## Abstract

Programmed cell death (PCD) is a process that plays a fundamental role in plant development and responses to biotic and abiotic stresses. Knowledge of plant PCD mechanisms is still very scarce and is incomparable to the large number of studies on PCD mechanisms in animals. Quick and accurate assays, e.g., the TUNEL assay, comet assay, and analysis of caspase-like enzyme activity, enable the differentiation of PCD from necrosis. Two main types of plant PCD, developmental (dPCD) regulated by internal factors, and environmental (ePCD) induced by external stimuli, are distinguished based on the differences in the expression of the conserved PCD-inducing genes. Abiotic stress factors, including heavy metals, induce necrosis or ePCD. Heavy metals induce PCD by triggering oxidative stress via reactive oxygen species (ROS) overproduction. ROS that are mainly produced by mitochondria modulate phytotoxicity mechanisms induced by heavy metals. Complex crosstalk between ROS, hormones (ethylene), nitric oxide (NO), and calcium ions evokes PCD, with proteases with caspase-like activity executing PCD in plant cells exposed to heavy metals. This pathway leads to very similar cytological hallmarks of heavy metal induced PCD to PCD induced by other abiotic factors. The forms, hallmarks, mechanisms, and genetic regulation of plant ePCD induced by abiotic stress are reviewed here in detail, with an emphasis on plant cell culture as a suitable model for PCD studies. The similarities and differences between plant and animal PCD are also discussed.

## 1. Introduction

Heavy metals are a harsh factor influencing plants that may disturb the biological processes in plant cells. In different regions of the world, soil is contaminated with heavy metals either naturally or anthropogenically. These areas are covered by plants known as metallophytes, taxa from different families that have developed tolerance to heavy metals through evolutionary processes (Box 1).

By inducing the generation of reactive oxygen species (ROS) and by direct effects, metal toxicity results in the inhibition of plant growth, necrosis, chlorosis, disturbed photosynthesis, and increased membrane permeability in cells [1]. Homeostasis is no longer maintained in plant cells under the influence of a certain threshold of stress factors due to disturbances in metabolic pathways, which may ultimately lead to cell death [2].

Cell death is categorized as programmed cell death (PCD) driven by regulated intracellular signals and nonprogrammed cell death as accidental cell death, including necrosis. Abiotic factors, including heavy metals, might affect cells and lead to necrosis or PCD [3,4]. Although necrosis is a common effect of many toxic stimuli, many authors have shown that heavy metals also lead to PCD [5,6,7,8]. PCD is a genetically controlled process coordinating the elimination of selected unnecessary cells and is considered one of the defense strategies of cells against biotic and abiotic stresses [4]. Plant PCD is separated into developmental PCD (dPCD) induced by internal signaling and environmental PCD (ePCD) influenced by external factors based on the expression of conserved PCD-inducing genes. Most of the dPCD-regulated genes are not expressed in ePCD [9]. The mechanisms and roles of proteases in dPCD are more well-known than the mechanisms of ePCD, particularly the pathways affected by abiotic factors [10,11].

Currently, knowledge of the execution processes leading to PCD in plants is very scarce. In animal cells, biochemical PCD events and molecular pathways are well characterized, and the caspase family of cysteine proteases plays the main role in the activation and execution of PCD [12]. Although typical caspase homologs have not been described in plants, enzymes with caspase-like activity have been identified [13,14]. In plants, cysteine proteases with caspase-like activity, particularly vacuolar processing enzymes (VPEs) and papain-like cysteine proteases (PLCPs), play the main roles in the PCD pathway [14,15]. VPEs are implicated in PCD during the hypersensitive response (HR) to pathogens and have enzymatic properties similar to animal caspase-1 [16]. PLCPs are involved in plant immunity, development, senescence, and abiotic stress responses [17,18].

The regulatory pathway of PCD as a tolerance strategy to environmental stimuli in specific plant cells must be clarified. In this review, we focused on several aspects of PCD in plants, including the current knowledge of the molecular mechanism of this process, developmental, and environmental PCD, and the differences between plant PCD and apoptosis in animals, with a special emphasis on PCD induced by heavy metals in plant cells.

Box 1 **Sources of heavy metals in the soil and heavy metal-tolerant plants.**

Soil may be contaminated with heavy metal(loid)s, e.g., lead (Pb), chromium (Cr), arsenic (As), zinc (Zn), cadmium (Cd), copper (Cu), cobalt (Co), nickel (Ni), manganese (Mn), mercury (Hg), or tungsten (W). These chemicals could have natural origin from pedogenic or lithogenic sources (ores), and the levels of bioavailable heavy metals are typically low and rarely toxic [19]; alternatively, the metals may be derived from human activity. The anthropogenic sources of heavy metals are mainly industry, mine tailings, leaded gasoline and paints, spillage of petrochemicals, wastewater sludges, pesticides or atmospheric deposition [20]. Heavy metals of anthropogenic origin tend to be more toxic to living organisms because they are usually more mobile and bioavailable than metals of natural origin [21]. The accumulation of heavy metals varies widely in soils due to the differences in the geochemical composition of the rocks and the intensity of soil-forming processes, which result in differences in the total and available concentrations of heavy metals [22]. Heavy metals differ significantly in their mobility and bioavailability (phytoavailability) to plants. Metal-soil physicochemical interactions affected by the organic matter content and pH dependence may influence heavy metal bioavailability [23]. Zn, Ni, and Cd are considered highly mobile heavy metals, whereas Cu, Cr, and Pb are strongly absorbed by the soil. Living organisms are affected only by the bioavailable fraction of the total heavy metal reservoir in the soil. Unlike organic contaminants that are oxidized and degraded by microbes, most heavy metals do not undergo degradation and remain in the soil for a long time [24].

Areas polluted with heavy metals are inhabited by plant species with a high tolerance to heavy metals, which is acquired through evolutionary processes. Only tolerant taxa that develop biochemical and physiological mechanisms against heavy metals are capable of living, growing and reproducing sexually at polluted sites [25]. Plants colonizing metalliferous areas that are tolerant to heavy metals are called metallophytes. They are found in over 34 unrelated plant families and are most frequent in the Asteraceae, Brassicaceae, Caryophyllaceae, Plumbaginaceae, Poaceae, and Violaceae families [26]. Soils colonized by metallophytes are classified based on the content of dominant heavy metals. Depending on the soil type (calamine—Zn/Pb, serpentine—Ni/Cr/Fe/Mg, cupriferous—Cu, arsenic—As), the floristic composition is different [27].

## 2. Developmental Versus Environmental PCD in Plant Cells—Genetic Regulation of ePCD

Developmental PCD in plants, which is regulated by internal factors, is associated with the reproduction, growth, or specialization (e.g., embryogenesis, organ shaping, aerenchyma formation, xylem formation, death of root cap or cork cells, or tapetum layer) and senescence of plant organs [11,28]. In contrast, ePCD is an adaptive response of plants to external stimuli. Some morphological and biochemical features, such as calcium signaling, generation of ROS or induction of VPE activity, are similar in dPCD and ePCD [29]. However, genes associated with PCD are differentially regulated in dPCD and ePCD, allowing the separation of these two types of PCD [9]. Stress induced by in vitro culture, mostly long-term cultivation and exposure to plant growth regulators, activates PCD-related genes and initiates the PCD process [30,31,32].

**Figure 1:**
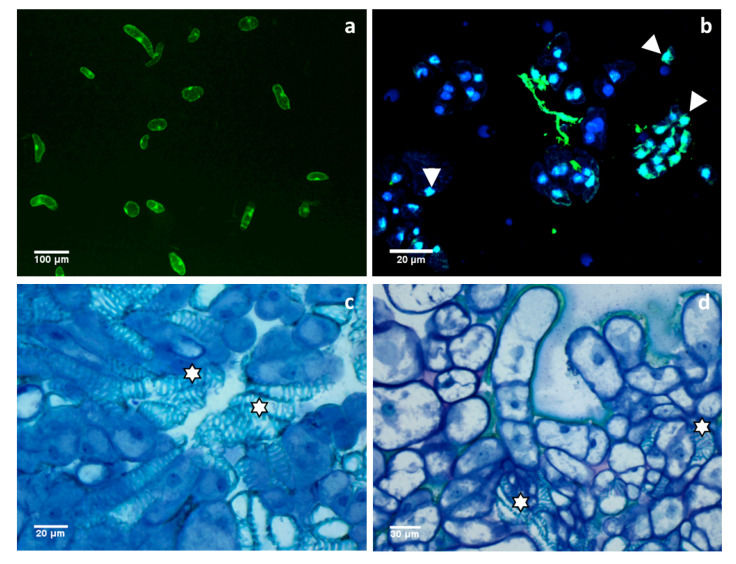
Cells and tissues of *Viola tricolor* cultured in vitro. Suspended cells (**a**), microcallus (**b**) and callus (**c**,**d**). (**a**)—viable cells after fluorescein diacetate staining. (**b**)—TUNEL-positive nuclei emitting light yellow-green fluorescence (arrowheads) in cells treated with Pb for 72 h. (**c**,**d**)—xylem vessels with lignified cell wall thickening among other callus cells as a result of dPCD (stars). Note the variation in size and shape of callus cells. (**c**,**d**)—Sections of calli stained with toluidine blue. Based on Sychta et al. [18], new photos were obtained from the private collection of K. Sychta.

In plants cultured in vitro, both dPCD and ePCD lead to cell death because different factors that initiate programmed mechanisms potentially affect suspended cells or tissues. One of the major dPCD events in cells cultured in vitro is xylogenesis—xylem formation stimulated by plant growth regulators [33,34], which has been documented in xylogenic zinnia (*Zinnia elegans*) cell suspension cultures [35] and in suspended cells of *Viola tricolor* cultured in vitro (Figure 1). Developmental PCD during xylem formation in *Arabidopsis* is controlled genetically by specific genes, such as VASCULAR-RELATED NAC-DOMAIN6 and VASCULAR-RELATED NAC-DOMAIN7, SOMBRERO and NAC (NO APICAL MERISTEM, ARABIDOPSIS TRANSCRIPTION ACTIVATOR FACTOR, and CUP-SHAPED COTYLEDON) transcription factors [11,36,37]. PCD in tracheary element differentiation during in vitro culture is associated with microtubule depolymerization and a decrease in the F-actin density [38,39]. Crucial functions triggering dPCD include Ca^2+^, ROS, pH, or ethylene signaling [29,40,41]. Cell suspension culture also provides a suitable model system for the investigation of senescence-related dPCD because cells in suspension have a determined cell cycle [42]. In this process, ORESARA1 is gene regulating senescence in tissues cultured in vitro [43].

Environmental PCD is directly or indirectly induced by external biotic (different types of pathogens) or abiotic (e.g., salinity, flooding, UV, drought, temperature, or heavy metals) signals [13,18,44]. One of the best-known types of ePCD is HR, a combination of resistance and cell death, which is activated during plant cell pathogen invasion [45]. During plant culture in vitro, the ingredients of the artificial medium for growth, particularly compounds such as mineral substances, vitamins, sugars, amino acids, or plant growth regulators as well as biotic or abiotic factors, lead to the mitochondrial oxidative burst and consequently to ePCD [4,46,47,48]. High concentrations of cytokinins during in vitro culture inhibit cell division and induce ePCD in *Daucus carota* and *Arabidopsis thaliana* cells. Interestingly, the addition of auxin, 2,4-dichlorophenoxyacetic acid or abscisic acid together with cytokinins inhibits cytokinin-induced PCD [49].

Some transcription factors may function as molecular switches in the regulation of PCD in plants triggered by environmental factors. The family of NAC transcription factors is involved in the activation of PCD in response to biotic stress and genotoxic damage and the regulation of HR. WRKY and MYB are activated by biotic stress to induce PCD, whereas ETHYLENE RESPONSIVE FACTOR regulates PCD following exposure to biotic and abiotic stimuli [50,51].

Similar to animals, the genes encoding Bax protein were reported to induce PCD in plants resembling the HR by the overexpression of ENHANCED DISEASE SUSCEPTIBILTY1 (EDS1) gene and increased ROS production [52]. However, ROS overproduction stimulated by biotic or abiotic stresses could initiate the expression of Bax Inhibitor-1, which slows down the cell death progression [53]. Likewise, autophagy related genes (*atg*) playing a role in autophagy, regulate HR as well [54]. Genes as LESION STIMULATING DISEASE1 (LSD1), PHYTOALEXIN DEFICIENT4 (PAD4), EDS1 are involved in PCD in plants under some environmental stresses. LSD1 regulates the expression of EDS1 and PAD4, genes controlling ROS, salicylic acid (SA) and ethylene production [55]. EDS1 has a role in the activation of HR, also being a nonspecific, stress-related protein [56,57]. LSD1 with LSD-ONE-LIKE1 (LOL1) may function as antagonistic transcriptional factors of PCD. High expression of LSD1 may antagonize cell death, whereas LOL1 overexpression will activate PCD [58]. Another gene family important in plant PCD activation is Receptor-like/Pelle kinases, among which a group of cysteine-rich Receptor-like/Pelle kinases play important role in PCD caused by environmental stimuli [59,60]. PCD induced by external stresses could be activated via ROS and mitogen-activated protein kinases (MAPKs) cascades. The prolonged activation of MAPKs results in ROS accumulation and finally activation of PCD process [61]. Cytochrome c (Cyt c) interacts with RESPONSIVE TO DESSICATION21 cysteine proteinase and GLYOXALASE II CYTOPLASMIC ISOZYME protein related with oxidative stress during PCD [62].

In PCD some signals are traveled in the process called retrograde signaling typically occurring between the chloroplasts or mitochondria and nuclei. ROS and jasmonic acid (JA) produced by peroxisomes induce PCD under light stress via OXIDATIVE SIGNAL-INDUCIBLE1 (OXI1)/MPK11/MPK13 pathway. Crosstalk between JA and SA, produced by chloroplasts along with DEFENDER AGAINST CELL DEATH1 (DAD1) and DAD2, regulates OXI1 expression. Another way is retrograde signaling pathway mediated by singlet oxygen under light stress. At low concentration it induces EXECUTER1-mediated PCD regulated by LSD1/LOL1. At high concentrations singlet oxygen oxidizes β-carotene to β-cyclocitral which induces singlet oxygen-related genes via METHYLENE BLUE SENSITIVITY1 leading to PCD [63]. 

Although caspases do not occur in plants, plant caspase-like proteases have been found. They are divided into two broad groups as legumain family cysteine endopeptidases, including VPEs and metacaspases, and subtilisin family serine peptidases, including saspases and phytaspases. All of them are involved into PCD caused by biotic and abiotic stimuli as well [64,65]. ARABIDOPSIS THALIANA METACASPASE1 (AtMC1) and AtMC2, genes encoding metacaspases, antagonistically control PCD in *Arabidopsis*. AtMC1 is a pro-PCD caspase-like protein, whereas AtMC2 inhibits AtMC1-depended PCD [66]. VPEs are activated in acidic environment provided by the vacuole and is also activated during HR and abiotic stresses [67]. Interestingly, saspases are not transcriptionally or translationally activated during stress response, but they are released into the intercellular space before the first signs of PCD. Saspases are incorporated into the signal cascade leading to Rubisco proteolysis [64]. Phytaspases are found in a wide variety of plant species and regulate PCD during biotic and abiotic stresses. However, plant caspase-like proteases, structurally distinct from animal caspases, have similar substrate cleavage specificities [65].

## 3. PCD in Plants and Apoptosis in Animals

True apoptosis is not observed in plant cells, although different abiotic factors cause morphological changes that are very similar to those occurring during animal cell apoptosis in a process called apoptotic-like PCD (AL-PCD) (Box 2). The features of AL-PCD are similar to animal cell apoptosis, such as cell shrinkage, DNA cleavage into smaller fragments, rigid cell walls without vacuole enlargement, increased mitochondrial activity, ROS production and Cyt c release, which were observed using immunochemistry, confocal and transmission (electron) microscopy in cells treated with abiotic stress factors [6,68,69,70,71,72,73,74]. Overall, hallmarks induced by different abiotic factors are very similar (reviewed in [75]).

Autolytic (vacuolar) PCD in plants is evidenced by the accumulation of lytic vacuoles and autophagosomes and a decrease in the cytoplasmic volume simultaneously with intact organelles and a normal turgor. The disruption of vacuolar membranes in autolytic PCD followed by the release of lytic enzymes leads to the degradation of cellular contents [74,76]. Autophagy, a process that is very similar to autolytic PCD involving autophagosomes, is required for nutrient remobilization during senescence, the clearance of damaged organelles and protein aggregates, and pathogen defense [77].

The HR is a manifestation of the defense response to environmental stimuli. Many HR features have been described in AL-PCD or autophagy, but ion fluxes, lipid peroxidation or cell wall reinforcement and the involvement of resistance genes (*R*-genes) commonly characterize the HR [78].

Although apoptosis is the most well-known type of PCD in animal cells, alternative mechanisms exist, such as autophagy that involves autophagosomes or necroptosis (programmed necrosis), which is morphologically similar to necrosis but regulated by specific molecules [12] (Box 2).

Box 2 **Plant and animal PCD—types, similarities and differences.**

**Differences:**

(A) animal PCD:

- contribution of cysteine proteases–caspases

- the presence of apoptotic bodies or cell protrusions and engulfment of these by other cells

- the presence of autophagosomes, autolysosomes and small lytic vacuoles

- involvement of Bcl-2 family proteins

Types of animal PCD:

- apoptotic: apoptosis, anoikis

- non-apoptotic: autophagy, entosis, methuosis, paraptosis, mitoptosis, parthanatos, ferroptosis, pyroptosis, NETosis, necroptosis

(B) plant PCD:

- contribution of proteases with caspase-like activity

- increase of nuclease and protease activity

- cell wall lignification, gelification, suberification

- increase of vacuolar volume (exception: AL-PCD)

- contribution of chloroplast by the ROS generation

- involvement of SA, JA and ethylene

Types of plant PCD: autophagy, apoptotic-like, hypersensitive response (HR), autolytic (vacuolar)

**Similarities:**

- chromatin condensation

- DNA fragmentation

- vacuolization of the cytoplasm

- the proteolytic cutting caused by caspases (animals) and caspase-like enzymes (plants) occurs always on Asp residue

- activation of PCD in mitochondria

- contribution of aliphatic polyamines, transglutaminases and hydrolases

- involvement of autophagy (atg) genes

- involvement of BAX protein

- involvement of MAPKs

- overproduction of ROS and NO

- Ca^2+^ accumulation

- loss of the mitochondrial integrity and Cyt c release

Based on [52,61,76,79,80].

## 4. Plant Cell Death under Abiotic Stress—PCD vs. Necrosis

Programmed cell death is a highly organized and regulated process, in contrast to necrosis. The shrinkage of the protoplast and nucleus, condensation of chromatin, cleavage of DNA and vacuolization are the basic features of PCD. Necrosis manifests as swelling of the protoplast and a loss of the integrity of the plasma membrane [4,81,82]. Furthermore, PCD is characterized by nonmorphological features, such as Cyt c release, massive ROS accumulation, and changes in the intracellular Ca^2+^ concentration and caspase-like enzyme activity [83,84,85].

The separation between PCD and necrosis based only on the morphological differences of cells is difficult, but several biochemical techniques enable researchers to distinguish PCD from necrosis (Box 3). The same factors may cause both PCD and necrosis, but the response of cells depends on the genotype/species and level of stress. Low stress levels lead to the repair of cell damage, moderate levels may initiate PCD and a very high level potentially leads to necrosis [86,87]. In soybean cells, a low concentration (8 mM) of hydrogen peroxide induces PCD with the traits of the HR, whereas a higher concentration (100 mM) leads to necrosis [88]. In tolerant poplar clones treated with ozone, the cells underwent PCD, whereas the same ozone concentration caused necrotic cell death in sensitive genotypes [89]. Therefore, PCD in tolerant genotypes might be a part of the signaling pathway activated as a defense response to stress.

Box 3 **How can PCD be identified in plant cells?**

Different histochemical staining methods that are commonly used to identify viable or nonviable cells do not recognize the causes of death, necrosis or PCD. Several biochemical indicators are useful tools to distinguish PCD from necrosis. A very common indicator of PCD in plant cells is the internucleosomal fragmentation of DNA, which is easily determined by the TUNEL assay that labels 3′-OH ends produced as a result of DNA fragmentation [18,71,90,91]. The early stage of PCD could be detected using a very fast and cost-effective DNA ladder assay, where specific endonucleases attack nuclear DNA, producing double-stranded DNA fragments with low molecular weights [92]. For single cells, a suitable and fast method is the comet assay (single-cell gel electrophoresis) that detects DNA degradation [93], but it should be used with caution because it may detect other processes related to DNA, e.g., alkali-labile sites or oxidized bases [94]. A reliable method for the detection of plant PCD is the analysis of caspase-like protease activity using caspase-like protease inhibitors (e.g., biotin-xVAD-fmk) and spectrophotometric methods, western blotting or ligand blotting [18,95]. Fluorescence resonance energy transfer was successfully used to monitor caspase-3-like activity in real time at the single-cell level in plant cells [96]. This method could also be useful for the visualization of signaling pathways during PCD in cells exposed to stressful factors.

## 5. PCD Induced by Heavy Metals in Plant and Animal Cells

**Figure 2:**
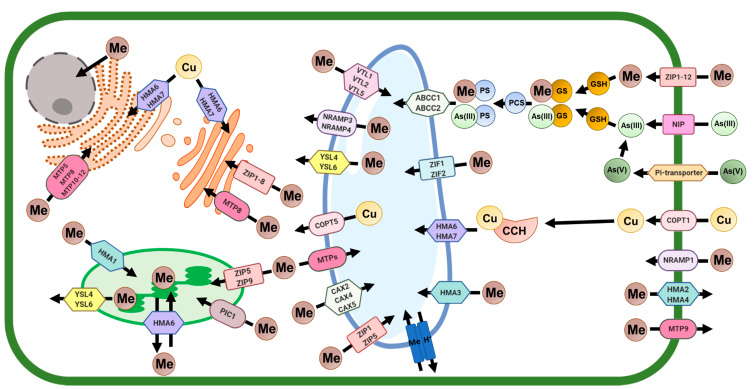
Summary of subcellular heavy metal transport in plant cells. Metal transporter proteins participating in heavy metal transport into and out of cellular organelles are presented. ABCC—ATP-binding cassette subfamily C protein; As—arsenic; CAX—Ca^2+^ exchanger; COPT—copper transporter; CCH—copper chaperone; Cu—copper; GS—glutathione conjugates; GSH—glutathione; HMA—heavy metal ATPase; Me—heavy metal; MTP—metal tolerance proteins; NIP—nodulin 26-like intrinsic protein; NRAMP—natural resistance-associated macrophage protein; PS—phytochelatin conjugates; PCS—phytochelatin synthase; Pi-transporter—phosphorus transporter; VTL—vacuolar-iron transporter-like; ZIF—zinc induced facilitator; ZIP—zinc regulated transporters; YSL—yellow stripe like (metal-nicotianamine transporter) (based on [97,98,99]).

**Figure 3:**
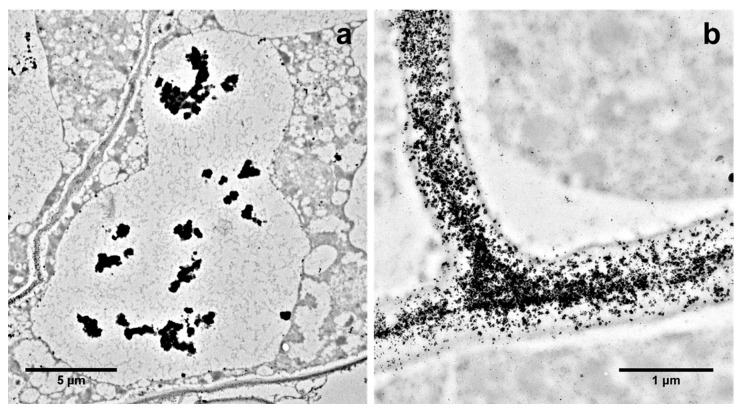
Pb deposition in the vacuoles (**a**) and cell walls (**b**) of *V. tricolor* cells treated with 2000 µM Pb for 72 h visible in transmission electron microscopy. Based on Sychta et al. [100], new photos were obtained from the private collection of K. Sychta.

Heavy metals easily enter plant cells and cell organelles via specific heavy metal transporters (Figure 2), causing disturbances in the functioning of cells and finally leading either to PCD or sequestration into the vacuole or deposition in the cell wall as a detoxification mechanism (Figure 3). In vitro culture, particularly cell suspension culture, is an excellent tool to study dPCD and ePCD regulation in plants due to the uniformity, accessibility and reduced complexity of this experimental model [81]. In plant cells, excess heavy metals cause disturbances in biological processes [1], and PCD is frequently activated upon the exposure of plant cells to heavy metals.

High concentrations of metals disrupt the antioxidant defense system in cells, which initiates the PCD process [101]. The exposure of tobacco BY-2 and *A. thaliana* cell suspension cultures to high concentrations of Cd induces PCD [7,102]. This process might be mediated by endogenous ethylene formation, which participates in Cd-induced PCD [103]. The cell death pathway could be determined by measuring the cell cycle phase in which abiotic factors are applied or act. The application of Cd in S and G2 phases was accompanied by internucleosomal DNA fragmentation and PCD (AL-PCD), whereas the application of this metal in M and G1 phases resulted in nonprogrammed cell death and necrosis [104].

**Figure 4:**
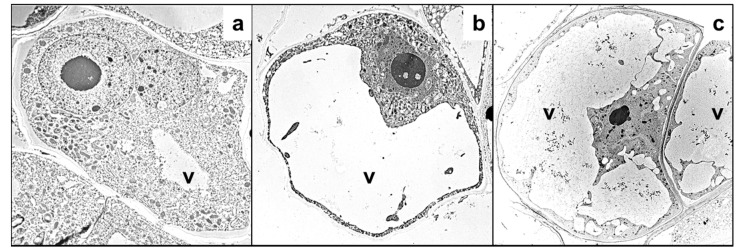
Vacuolization of *V. tricolor* cells visible in transmission electron microscopy. (**a**)—control cells. (**b**)—cells treated with 2000 µM Zn for 72 h. (**c**)—cells treated with 2000 µM Pb for 72 h. v—vacuole. Based on Sychta et al. [100], new photos were obtained from the private collection of K. Sychta.

*Vitis vinifera* cell cultures exposed to Ag showed a high frequency of cells undergoing PCD accompanied by ROS formation, caspase-3-like activity and degradation by the ubiquitin-proteasome system [72]. According to Poborilova et al. [105], Al_2_O_3_ lead to PCD via ROS and nitric oxide (NO) generation and that is executed by caspase-3-like enzyme activation in both a time- and dose-dependent manner. Interestingly, W induces PCD in plant cells through a mechanism that is not mediated by ROS production, as observed in PCD induced by other heavy metals. A low concentration of ROS is detected simultaneously with minimal mitochondrial and chloroplast degradation during PCD induced by W, the organelles in which ROS are commonly generated [106]. Nickel and Cr induce PCD with AL-PCD-type features, such as protoplast condensation with DNA degradation, in *Vicia faba* and *Allium cepa* root tip cells [107]. Cadmium promotes peroxisome proliferation, leading to a specific type of autophagy—pexophagy—as a rapid cellular response of *A. thaliana* cells to metal treatment [108]. Heavy metals induce vacuolar cell death in suspended *V. tricolor* cells treated with Zn or Pb, as identified by intense cell vacuolization (Figure 4), which was also observed in other plant species [3] (Table 1).

The cytotoxic effect of heavy metals is not only due to their excess concentrations but also due to their interactions with other elements present in the culture medium (reviewed in [131]). Boron, which alleviates Cd toxicity in *Brassica napus* by promoting the chelation of Cd onto root cell wall components [132], may exert the opposite effect. Boron deficiency significantly increased the PCD rate by activating ROS signaling pathways and oxidative damage in tobacco BY-2 cells treated with 150 µM HgCl_2_ or CdCl_2_ [71,90]. Additionally, a B deficiency alone significantly increased the frequency of TUNEL-positive cells, ROS production and the level of lipid peroxides, supporting the hypothesis that the deprivation of microelements might produce similar effects to their excess on plant cells.

Different toxins, including heavy metals, may induce apoptosis in animal cells (Box 4). Morphological and molecular differences in plant and animal PCD mechanisms have been identified, but some of the PCD features are shared by the two kingdoms, such as ROS generation, Cyt c release, Ca^2+^ accumulation, or the involvement of protease activity, which are presumably conservative traits. Likewise, MAPK playing a role in PCD induced by heavy metals in animal cells is also activated during heavy metal stress and PCD process in plant cells [61,133,134].

Box 4 **PCD mechanism induced by heavy metals is not unique in animal cells.**

Most heavy metals induce PCD in animal cells through oxidative stress via the generation of ROS, Ca^2+^ accumulation, upregulation of caspases, particularly caspase-3, or downregulation of the apoptosis regulators as B-cell lymphoma 2 (Bcl-2) and metallothionein, and p53 overexpression. Cadmium, Cr, and Ni are capable of inducing apoptosis along with DNA modifications and rearrangements [135]. Cadmium is known to induce apoptosis in various types of mammalian cells by inhibiting mitochondrial respiration followed by Cyt c release or caspase-3, 8, and 9 activation. Cadmium also induces caspase-independent PCD via endonucleases or calpain activity [136,137]. Arsenic induces apoptosis by promoting ROS generation, caspase-3 activation, calcium balance disorders or the depletion of intracellular glutathione, supported by the involvement of mitochondria with Cyt c release [138,139]. The transforming growth factor beta pathway is involved in the apoptosis process of liver cells under Cr stress [140]. Nickel induces PCD through oxidative stress caused by excess ROS production and the downregulation of Bcl-2 [135]. Arsenic induces PCD by activating the mitogen-activated protein kinase/extracellular signal-regulated kinase pathway, Beclin-1 and caspase-3, 8, and 9 [133]. Following Cu exposure, mitochondria are not involved in the activation of apoptosis through ROS generation. Copper reduces the content of X-linked inhibitor of apoptosis in cells, leading to the activation of the apoptosis process [135]. In cells treated with Pb, mitochondria play a crucial role in the induction of apoptosis by releasing Cyt c. Caspase activation leading to apoptosis is also induced by Pb exposure [141].

## 6. The Physiological Characteristics of PCD in Plant Cells under Heavy Metal Stress

Reactive oxygen species production in plant cells is stimulated by lipid signaling pathways, mainly by phospholipase C and D, producing phosphatidic acid under heavy metal stress [5,6,69]. Many studies have documented that ROS generated in plant cells under heavy metal stress play a crucial role in PCD (e.g., [4,71,72,90,142]). In mitochondria, which are involved in ROS production, overexpression of ALTERNATIVE OXIDASE 1a alleviates Al-induced PCD by maintaining mitochondrial function, providing insights into the role of these organelles in modulating the Al phytotoxicity mechanism [48,70]. Aluminum inhibits the electron transport chain upon entering mitochondria, leading to the accumulation of ROS and subsequently resulting in the rupture of the mitochondrial membrane and the release of Cyt c to the cytosol in *Arabidopsis* mesophyll protoplasts [48]. The amount of hydrogen peroxide produced by plasma membrane NADPH oxidase is not sufficient to trigger all deleterious effects of Cd on plant cells. Superoxide anion radical of mitochondrial origin is thus a key factor in Cd-induced cell death [143]. Salicylic acid has a role in the induction of Cd-induced PCD in tobacco cells. Cadmium ions increase the internal SA content and induce the SA-induced protein kinase signaling pathway, leading to PCD by inhibiting the antioxidant system [144].

Ethylene, the level of which is modulated by ROS formation and stimulates the redistribution of Ca^2+^, also participates in the onset of PCD [5,7]. The cellular response to Cd and Pb involves complex crosstalk between ROS, hormones (ethylene), NO, and Ca^2+^ [6,91,145]. Accordingly, NO modulates the content of phytochelatins (PCs), whose function is to bind heavy metals in the cytoplasm and to mediate their vacuolar sequestration [102]. This multifunctional gas also modulates Cd uptake in tobacco BY-2 cells, stimulating Cd-induced PCD [7]. However, researchers have not clearly determined whether Cd uptake is achieved by PC engagement, although the PC level is remarkably increased during the pre-PCD phase and reaches a limiting value at cultivation times coinciding with the PCD trigger in the presence of 150 µM Cd [146]. The NO burst occurred earlier than ROS accumulation, influenced Ca^2+^ effluxes and disrupted Ca homeostasis in *Nicotiana tabacum* cells treated with Pb [91]. The inhibition of Ca transients eliminated cell death in *A. thaliana* suspensions, highlighting the role of Ca in PCD [147]. In plant species growing in the presence of high Mg and low Ca concentrations (serpentine plant community), a greater percentage of cell death was induced by Ca^2+^ than by Mg^2+^, suggesting that serpentine plants are not able to regulate cell death rates when imbalances in intracellular Ca^2+^ homeostasis are present. A comparative analysis of the permeability of cell membranes to Ca^2+^ between serpentine and nonserpentine species is thus desired [73].

Further mechanisms involved in heavy metal induced PCD were described based on ultrastructural studies of suspended *Pisum sativum* roots under W stress, which induces vacuolar destructive PCD without ROS generation [106,109]. In this study and a study using suspended tobacco cells, PCD of plant cells under W and Cd stress was induced by the endoplasmic reticulum (ER) stress pathway triggered by unfolded protein production [106,148]. Cadmium-induced PCD stress was relieved by two ER chemical chaperones, 4-phenylbutyric acid and tauroursodeoxycholic acid, providing insights into this newly described mechanism of plant cell death upon exposure to heavy metals [148].

## 7. Regulation and Execution Processes Engaging Plant Proteases with Caspase-Like Activity during Plant PCD Induced by Heavy Metal Stress

Plant proteases regulate PCD by activating a cascade of protein degradation processes. The proteases in plants are divided into four major catalytic classes: cysteine, serine, aspartic threonine and metalloproteases. Typical caspases have not been described in plant cells; however, proteases with caspase-like activity play a role in the plant PCD process [14,149]. Many cysteine proteases (e.g., PLCPs, caspase-like proteases, metacaspases, the proteasome or VPEs) are involved in plant PCD [15,123]. Various experiments using synthetic caspase substrates revealed caspase-like activity in plant systems [13]. Plant caspase-like proteases appear to be ancestors of animal caspases.

Broad-range protease inhibitors successfully inhibit PCD induced by heavy metals [18,150]. *V. vinifera* cells exposed to 10 µM Ag^+^ underwent PCD accompanied by the activation of caspase-3-like activity and generation of ROS. The addition of DEVD-CHO (a caspase-3 inhibitor) significantly decreased the number of dead Ag-treated cells [72]. After the addition of Al to the cell suspension culture of the tobacco cell line BY-2, the activity of VPEs was increased simultaneously with the collapse of vacuoles and loss of plasma membrane integrity, leading to vacuolar cell death [151]. Interestingly, Al-induced PCD in peanut (*Arachis hypogaea*) root tip cells is activated by a number of caspase-like proteases, but the crucial enzyme in this process is a caspase-3-like enzyme, with the highest activity observed after Al addition [117]. The same finding was described in *Triticum aestivum* root tip cells, where caspase-3-, caspase-8-, and caspase-9-like activities were detected after the addition of Al_2_O_3_ [120]. Metacaspase AhMC1 induces PCD in peanut root tip cells after an Al treatment, and its overexpression reduces the resistance of peanuts to this metalloid [118]. The relationship between NO, MPK6 and caspase-3-like activation in PCD induced by Cd exposure was documented. NO promoted Cd-induced PCD by inducing caspase-3-like activation via the MPK6 in *A. thaliana* cells [110].

New discoveries include the detection of PLCP activity in suspended *V. tricolor* cells treated with high concentrations of Zn or Pb. Additionally, the direct application of E64-d (a specific PLCP inhibitor) to the cell suspension culture with these heavy metals successfully inhibited the PCD process [18]. The proteases with caspase-like activity involved in plant PCD associated with heavy metal stress are summarized in Table 2.

## 8. Conclusions and Future Perspectives

The regulation and execution processes of PCD, particularly the processes induced by abiotic factors such as heavy metals, remain *terra incognita* (Box 5), although some of the biochemical, molecular and morphological mechanisms are known. In this review, we have focused on the PCD process, mechanisms, hallmarks induced by heavy metals, and other abiotic factors in plant cells, including proteases with caspase-like activity in PCD and the mechanism of PCD inhibition. PCD is a crucial process in plant development, senescence, or immunity and plays an important role in the plant stress response. The plant cell PCD mechanisms and features are also compared with apoptosis in animal cells. The processes and biochemical and molecular pathways of plant PCD induced by abiotic stress are very important for understanding the tolerance/resistance of plants to abiotic factors, enabling plant tolerance to be increased in the future by manipulating the inhibition of PCD. In a globally changing environment with climate change, tolerant genotypes/species are highly desirable.

Box 5 **Outstanding questions.**

1. Is the molecular mechanism underlying the inhibition of PCD in plant cells under heavy metal stress more effective in cells from metallophyte species than in cells from nonmetallophyte species?

2. Is PCD in plant cells under heavy metal stress “true PCD” (an adaptation process to biotic or abiotic stress factors resulting in cell death) or “ersatz” PCD (intrinsic to the cell, but the death pathway itself has not been selected for), as described by Durand and Ramsey [153]?

3. Are cells/genotypes tolerant to multiple abiotic stresses (in vitro: culture environment, plant growth regulators, heavy metals; in nature: drought stress, strong winds, insolation, heavy metals in soil, etc.) due to the ability to suppress cell death and maintain growth by tuning hormone signaling and primary and secondary metabolism?

4. Are cyclotides (small plant cyclic peptides synthesized in some plant families), a plant host defense system protecting against pathogens and pests [154], a part of the plant cell-specific detoxification mechanism for heavy metals by capturing metals?

5. Is cell tolerance under heavy metal stress temporal, epigenetically regulated, or heritable to the next generations via DNA or chromatin modifications and small RNA-based mechanisms that regulate the expression of stress-responsive genes [155]?

## Figures and Tables

**Table 1:** PCD induced by different heavy metals in different type of cells of plant species.

Heavy Metal	Plant Species	Type of Cells	Reference
Tungsten	*Pisum sativum* L.	Root tip cells	[106,109]
Silver	*Vitis vinifera* L.	Cell suspension culture	[72]
Cadmium	*Arabidopsis thaliana* (L.) Heyhn.	Cell suspension culture Cells of young seedlings	[102,110]
	*Nicotiana tabacum* L.	Cell suspension culture	[111,112]
	*Lycopersicon esculentum* Mill.	Cell suspension culture	[5]
	*Lupinus luteus* L.	Root tip cells	[8]
	*Genipa americana* L.	Root tip cells Leaf cells	[113]
	*Malus hupehensis* Rehd.	Root tip cells	[114]
	*Populus alba* L.	Cell suspension culture	[115]
Aluminum	*Lycopersicon esculentum* Mill.	Cell suspension culture	[6]
	*Arachis hypogaea* L.	Root tip cells	[116,117,118]
	*Nicotiana tabacum* L.	Cell suspension culture	[119]
	*Arabidopsis thaliana* (L.) Heyhn.	Mesophyll protoplasts	[48]
	*Triticum aestivum* L.	Root tip cells	[120]
Zinc	*Nicotiana tabacum* L.	Cell suspension culture	[121]
	*Viola tricolor* L.	Cell suspension culture	[18]
	*Solanum nigrum* L.	Root tip cells	[122]
Lead	*Nicotiana tabacum* L.	Cell suspension culture	[123]
	*Viola tricolor* L.	Cell suspension culture	[18]
Copper	*Oryza sativa* L.	Root tip cells	[124]
	*Cucumber sativus* L.	Root border cells	[125]
	*Vigna radiata* (L.) R. Wilczek	Cells of seedlings	[126]
Cobalt	*Solanum melongena* L.	Root cells	[127]
Mercury	*Nicotiana tabacum* L.	Cell suspension culture	[71]
Nickel	*Lycopersicon esculentum* Mill.	Root cells of seedling	[128]
Nickel and Chromium	*Allium cepa* L. *Vicia faba* L.	Root tip cells	[107]
Iron	*Raphanus sativus* L.	Root cells	[129]
	*Allium cepa* L.	Root cells	[130]

**Table 2:** The classes of proteases with caspase-like activity involved in programmed cell death (PCD) induced by different heavy metals in different plant species.

Heavy Metal	Type of Protease	Plant Species	Reference
Tungsten	Caspase-1-like	*Pisum sativum* L.	[109]
Silver	Caspase-3-like	*Vitis vinifera* L.	[72]
Cadmium	Caspase-3-like	*Arabidopsis thaliana* (L.) Heyhn.	[110]
	Caspase-1-like	*Lycopersicon esculentum* Mill.	[5]
Aluminum	VPE	*Nicotiana tabacum* L.	[151]
	Caspase-1-like	*Arachis hypogaea* L.	[117]
	Caspase-2-like		
	Caspase-3-like		
	Caspase-4-like		
	Caspase-5-like		
	Caspase-6-like		
	Caspase-8-like		
	Caspase-9-like		
	Metacaspase AhMC1	*Arachis hypogaea* L.	[118]
	Caspase-3-like	*Triticum aestivum* L.	[120]
	Caspase-8-like		
	Caspase-9-like		
	Caspase-3-like	*Secale cereale* *Triticosecale wittmack* *Hordeum vulgare* *Avena sativa*	[152]
	Caspase-8-like		
	Caspase-9-like		
	Caspase-3-like	*Nicotiana tabacum* L.	[105]
	Caspase-3-like	*Arabidopsis thaliana* L.	[48]
Zinc	Papain-like cysteine protease	*Viola tricolor* L.	[18]
Lead	Papain-like cysteine protease	*Viola tricolor* L.	[18]
Nickel	Caspase-3-like	*Lycopersicon esculentum* Mill.	[128]
Nickel and chromium	Caspase-3-like	*Allium cepa* L. *Vicia faba* L.	[107]

## Data Availability

No new data were created or analyzed in this study. Data sharing is not applicable to this article.

## Abbreviations

AL-PCD	apoptotic-like programmed cell death
*atg*	autophagy related genes
AtMC	arabidopsis thaliana metacaspase
Bcl-2	B-cell lymphoma 2
Cyt c	cytochrome c
DAD	defender against cell death
EDS1	enhanced disease susceptibility 1
ER	endoplasmic reticulum
HR	hypersensitive response
LOL1	LSD-one-like 1
LSD1	lesion stimulating disease 1
MAPK/MPK	mitogen-activated protein kinase
NO	nitric oxide
OXI1	oxidative signal-inducible 1
PAD4	phytoalexin deficient 4
PC	phytochelatin
PCD	programmed cell death
PLCP	papain-like cysteine protease
R-genes	resistance genes
ROS	reactive oxygen species
SA	salicylic acid
TUNEL	terminal deoxynucleotidyl transferase dUTP nick end labeling
VPE	vacuolar processing enzyme
